# Metabolic Syndrome in Patients with Non-alcoholic Fatty Liver Disease: A Community Based Cross-sectional study

**DOI:** 10.7759/cureus.4099

**Published:** 2019-02-19

**Authors:** Mukesh S Paudel, Awadhesh Tiwari, Amrendra Mandal, Barun Shrestha, Paritosh Kafle, Baikuntha Chaulagai, Sudhamshu KC

**Affiliations:** 1 Gastroenterology, Lumbini City Hospital, Patan, NPL; 2 Radiology, Lumbini Medical College and Teaching Hospital, Palpa, NPL; 3 Internal Medicine, Interfaith Medical Center, Brooklyn, USA; 4 Gastroenterolgy, Chitwan Medical College, Bharatpur, NPL; 5 Hepatology, National Academy of Medical Sciences, New Delhi, USA

**Keywords:** metabolic syndrome, non-alcoholic fatty liver disease, obesity, waist circumference

## Abstract

Background

Non-alcoholic fatty liver disease (NAFLD) is the deposition of fat inside liver cells in the absence of secondary causes. It is considered as a hepatic complication of metabolic syndrome. The metabolic syndrome consists of dyslipidemia, hypertension, diabetes, and obesity. This study aims to determine the prevalence of metabolic syndrome in Nepalese patients with NAFLD from mid-Western part of Nepal.

Method

This was a descriptive cross-sectional study. Three different sites were chosen in and around Butwal sub-metropolitan city of Rupandehi district, Nepal. A one-day health camp for the screening of fatty liver disease by ultrasonography (USG) was conducted at these sites. Participants with fatty liver were then classified into three grades by USG and the presence of metabolic syndrome was assessed by the National Cholesterol Education Program Adult Treatment Panel III (NCEP-ATPIII) criteria.

Results

A total of 385 participants with NAFLD were evaluated. Presence of metabolic syndrome by NCEP-ATPIII criteria was found to be in 57.6% participants; whereas, at least one component of metabolic syndrome was found in 91.4% of participants with radiologic features of fatty liver. Higher proportion of patients with NAFLD were males. Increased waist circumference followed by low high-density lipoprotein (HDL) level were the most common components of metabolic syndrome in participants with NAFLD.

Conclusions

Metabolic syndrome is common in Nepalese community patients with NAFLD.

## Introduction

Non-alcoholic fatty liver disease (NAFLD) has emerged as an important cause of chronic liver disease. It is defined as the accumulation of fat inside the liver cells and the absence of secondary causes of hepatic fat accumulation (alcohol, drugs, viruses, and others) [1]. Evidence of hepatic steatosis is established either by imaging or histology. The metabolic syndrome consists of dyslipidemia, hypertension, diabetes, and obesity [2]. NAFLD is considered as a hepatic complication of metabolic syndrome [3].

There is shown to be an increased prevalence of NAFLD in people with metabolic syndrome and vice versa. NAFLD has a reported prevalence ranging from 20% to 30% depending on the studied populations [4]. The prevalence of metabolic syndrome in NAFLD is shown to be between 50% to 67% in various studies [5-6].

There is a paucity of data on the association of metabolic syndrome with NAFLD from our part of the world. The aim of the current study was to determine the prevalence of metabolic syndrome in people with NAFLD.

## Materials and methods

This was a descriptive cross-sectional study. It was conducted from August 1, 2018 to January 13, 2019. Ethical approval was obtained from the Institutional Review Committee of Lumbini Medical College and Teaching Hospital (IRCLMC) prior to the commencement of the study. Informed written consent was taken from the participants. The study population consisted of participants of a one-day health camp conducted on separate days in Pratappur village development committee (VDC) of Nawalparasi district, Shankarnagar in Tilottama Municipality and Belbas of Butwal sub-metropolitan city.

The sample size was calculated using the following formula: n=Z2P (1-P)/d2; where n is the sample size, Z is the statistic corresponding to level of confidence, P is expected prevalence and d is precision. Assuming Z= 95%, prevalence of metabolic syndrome=50% and d=0.05, the sample size was calculated to be 384. A non-probability convenient sampling method was used.

All participants who were 18 years of age and above were screened in these health camps for fatty liver by portable ultrasonography (USG) machine (ATNL/51353A, 3.5 MHz convex probe) by a radiologist with more than 10 years of experience in abdominal USG. The exclusion criteria consisted of regular consumption of alcohol >2 units (20 grams) per day, intake of drugs known to cause fatty liver, and history of viral hepatitis, Autoimmune hepatitis, Primary Biliary Cholangitis or Wilsons disease. All eligible participants were then invited to Lumbini City Hospital, Butwal for further evaluation.

A second ultrasound of abdomen was done by the same radiologist and grading of fatty liver disease was done using USG machine from Toshiba Aplio 400 series (Toshiba, Tokyo, Japan) and convex abdominal probe 3.5 MHz. Fatty liver was classified into three grades [7]. Grade I: increase in echogenicity of liver only, Grade II: echogenic liver obscures the echogenic walls of portal vein branches, and Grade III: echogenic liver not only obscures portal vein branches but also the outline of the diaphragm. Participants underwent detailed physical examination and laboratory examination using Biosystems BTS-350 (Biosystem SA, Barcelona, Spain) and colorimetry. A measuring tape was used to measure waist circumference at the midpoint between the costal margin and anterior superior iliac spine. Any potential source of bias was reduced by using the same instruments in each participant. According to the National Cholesterol Education Program Adult Treatment Panel III (NCEP-ATPIII) definition, metabolic syndrome was said to be present if three or more of the following five criteria were met: waist circumference over 40 inches (men) or 35 inches (women), blood pressure over 130/85 mmHg, fasting triglyceride (TG) level over 150 mg/dl, fasting high-density lipoprotein (HDL) cholesterol level less than 40 mg/dl (men) or 50 mg/dl (women) and fasting blood sugar over 100 mg/dl [8].

Data were entered in Microsoft Excel. Continuous data were expressed as mean and standard deviation. Categorical data were expressed as numbers and percentage. SPSS software version 22 (SPSS Inc., Chicago, IL, USA) was used to analyze the data. We used unpaired t-test for the analysis of continuous variables and chi-square test or Fisher's exact test for categorical variables. Quantitative variables were not grouped during analysis. P value of <0.05 was considered statistically significant.

## Results

**Figure 1:**
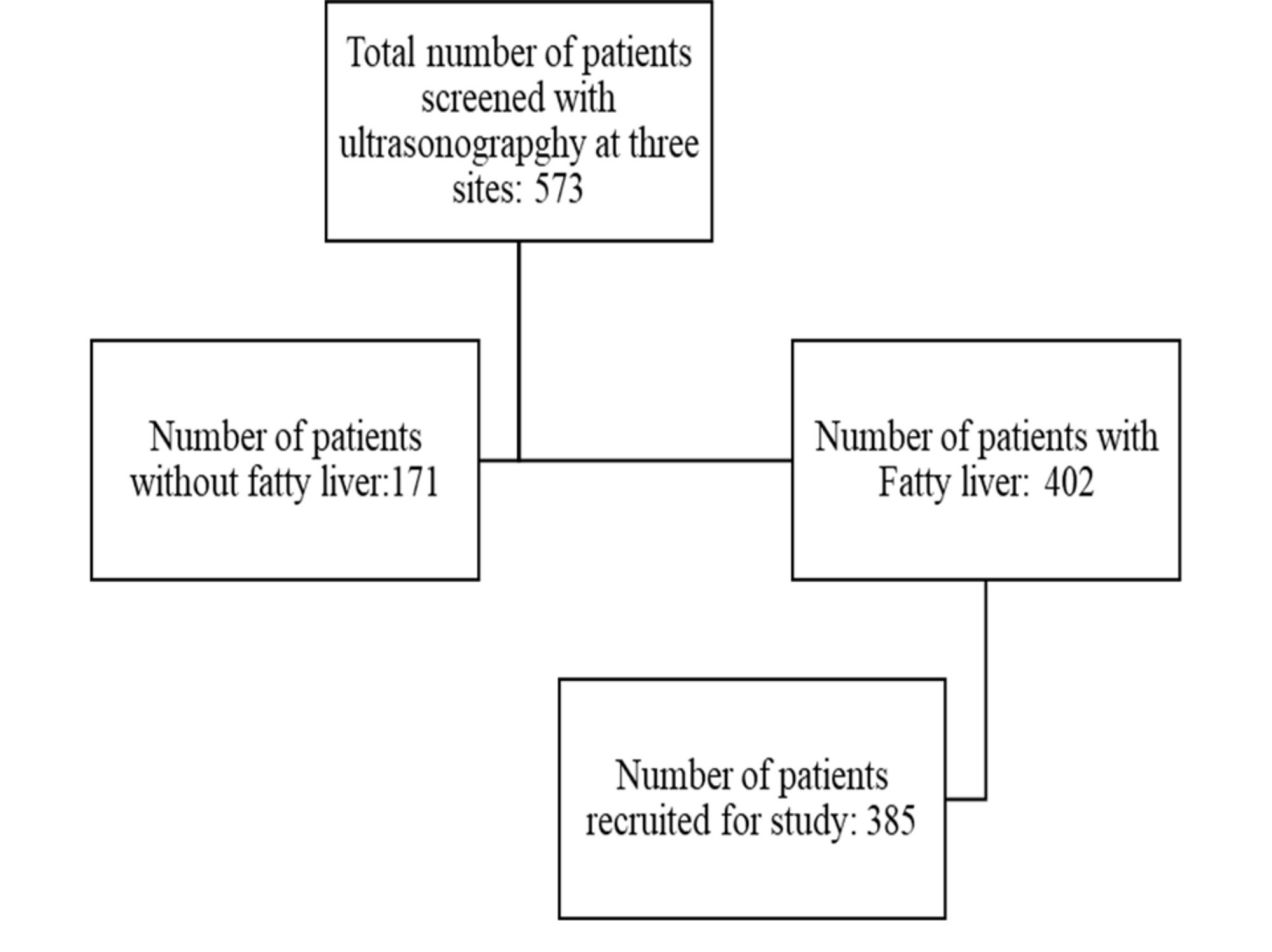
Process of recruitment of participants in the study

A total of 573 participants underwent screening USG at three different health camps. Four hundred and two patients were found to have fatty liver by screening USG. The processes of recruitment loss to follow up is shown in Figure 1.

Of the 385 patients that were evaluated at the Lumbini City Hospital and found to have fatty liver by USG, 209 were males and 176 were females. The average age of participants was 44.31± 12.58 years. Across all grades of fatty liver, the difference in average age of males and females was statistically significant as shown in Table 1.

**Table 1:** Frequency and mean ± standard deviation (SD) of the age of the participants based on different grades of NAFLD NAFLD: non-alcoholic fatty liver disease

Variable	Grade	Males		Female		p-value
		Frequency	Age (years)	Frequency	Age (years)	
NAFLD	I	131	37.17±7.70	121	46.48±13.17	<0.0001**
	II	70	43.49±11.91	53	54.45±10.2	<0.0001**
	III	8	61.13±4.55	2	74.00±5.66	0.0085**

Metabolic syndrome was present in 222 (57.66%) participants, whereas at least a single component of metabolic syndrome was present in 352 (91.4%) participants. All five components of metabolic syndrome were present in 41 (10.64%) participants.

Grade I fatty liver was seen in 65.4% (n=252, N=385) participants, Grade II fatty liver in 31.9% (n=123, N=385), and Grade III fatty liver in 2.5% (n=10, N=385).

Of all the components of metabolic syndrome, most of the patients had increased waist circumference followed by low HDL level as shown in Table 2.

**Table 2:** Proportion of patients with various components of metabolic syndrome

Components of metabolic syndrome	Number of patients n(%)
High blood pressure	197 (51.1)
Raised blood glucose	176 (45.7)
Increased waist circumference	258 (67.0)
Raised serum triglycerides level	232 (60.2)
Low HDL level	239 (62.0)

## Discussion

A total of 385 participants with NAFLD were evaluated in this community-based study and prevalence of metabolic syndrome by NCEP-ATPIII criteria was found to be in 57.6% (n=222) whereas at least one component of metabolic syndrome was found in 91.4% (n=352) participants with radiologic features of fatty liver.

In a population-based study on 3,613 patients with NAFLD in the United States, the average age of participants was 43 years which is similar to our study [9]. In a cross-sectional study from Japan, the maximum number of patients with NAFLD were shown to be in the age range of 40 to 49 years [10]. It has also been suggested that males may have a higher prevalence of NAFLD as compared to females and this difference may be related to hormonal factors [11]. Hormonal factors may also be responsible for the difference in age of occurrence of three grades of NAFLD between males and females. Our study also showed a higher proportion of patients with NAFLD to be males (n=209, 54.28%).

Prevalence of metabolic syndrome in the aforementioned population-based study was shown to be in 67% of patients [9]; 66% of males and 65% of females with NAFLD were found to have metabolic syndrome by NCEP-ATPIII criteria in another study [6]. Studies have shown that 90% of patients with NAFLD have at least one component of metabolic syndrome and 33% have all features of metabolic syndrome [12]. All five components of metabolic syndrome were present in 41 (10.64%) of our study participants. In another hospital-based study from Nepal, the prevalence of metabolic syndrome in patients with NAFLD was found to be 13.6% with NCEP-ATPIII criteria and 30.1% with the International Diabetes Federation (IDF) criteria. The difference in the prevalence of metabolic syndrome from our study may be due to the difference in the inclusion criteria [13].

Our study had a slightly higher proportion of patients with Grade I fatty liver as compared to another study from Nepal [14]. The difference may be due to the retrospective nature of their study and the fact that ours was conducted after screening patients in the community.

Metabolic syndrome is an interplay of various factors like obesity, dyslipidemia, dysglycemia, and raised blood pressure [15]. In patients who have NAFLD, the prevalence of obesity ranges from 30%-100% [16]. Increased waist circumference was seen in 258 (67%) of participants in our study. Also, the presence of type 2 diabetes mellitus is seen in 10% to75% of patients with NAFLD [12]. Only 176 (45.7%) of our study participants with NAFLD had raised blood glucose or diabetes mellitus. Presence of increased serum triglyceride level is seen in 64% and low HDL levels are seen in 30% to 42% of patients with NAFLD [17]. Two hundred and thirty-nine (60%) patients in our study had raised serum triglyceride and low HDL levels each.

Although NAFLD and metabolic syndrome are closely interrelated, there may be some genetic conditions in which both may not co-exist [15]. We did not evaluate for any genetic polymorphisms for the presence of fatty liver such as Patatin-like phospholipase domain-containing protein 3 (PNPLA3) in our study; and found that 163 (42.4%) of patients with NAFLD didn’t have metabolic syndrome.

Our study had few limitations. We have not looked into the prevalence of underweight patients in those with NAFLD and also didn't use liver biopsy to categorize benign fatty liver vs. non-alcoholic steatohepatitis. Further study may be needed to stratify these higher risk groups in order to treat promptly.

## Conclusions

There is a high prevalence of metabolic syndrome in the Nepalese community with NAFLD. Considering the fact that components of metabolic syndrome are also risk factors of cardiovascular disease, we recommend screening for such factors in patients with seemingly benign finding of fatty liver in USG of the abdomen.
